# Regulation of ATP hydrolysis by the ε subunit, ζ subunit and Mg-ADP in the ATP synthase of *Paracoccus denitrificans*

**DOI:** 10.1016/j.bbabio.2020.148355

**Published:** 2021-03-01

**Authors:** Owen D. Jarman, Olivier Biner, Judy Hirst

**Affiliations:** The Medical Research Council Mitochondrial Biology Unit, University of Cambridge, The Keith Peters Building, Cambridge Biomedical Campus, Hills Road, Cambridge CB2 0XY, UK

**Keywords:** *P. denitrificans*, F_1_F_O_-ATP synthase, ATP hydrolysis, ε subunit, ζ subunit, Mg-ADP inhibition

## Abstract

F_1_F_O_-ATP synthase is a crucial metabolic enzyme that uses the proton motive force from respiration to regenerate ATP. For maximum thermodynamic efficiency ATP synthesis should be fully reversible, but the enzyme from *Paracoccus denitrificans* catalyzes ATP hydrolysis at far lower rates than it catalyzes ATP synthesis, an effect often attributed to its unique ζ subunit. Recently, we showed that deleting ζ increases hydrolysis only marginally, indicating that other common inhibitory mechanisms such as inhibition by the C-terminal domain of the ε subunit (ε-CTD) or Mg-ADP may be more important. Here, we created mutants lacking the ε-CTD, and double mutants lacking both the ε-CTD and ζ subunit. No substantial activation of ATP hydrolysis was observed in any of these strains. Instead, hydrolysis in even the double mutant strains could only be activated by oxyanions, the detergent lauryldimethylamine oxide, or a proton motive force, which are all considered to release Mg-ADP inhibition. Our results establish that *P. denitrificans* ATP synthase is regulated by a combination of the ε and ζ subunits and Mg-ADP inhibition.

## Highlights

• Inhibition by Mg-ADP prevents ATP hydrolysis in *P. denitrificans*.
• Deleting the C-terminus of the ε subunit did not substantially activate hydrolysis.
• Deleting the ε-CTD did not compromise ATP synthesis.
• *P. denitrificans* ATP synthase is regulated by the ε and ζ subunits and Mg-ADP.

## 1 Introduction

F_1_F_O_-ATP synthase, a ubiquitous enzyme found in the inner membranes of mitochondria and chloroplasts and the cytoplasmic membranes of bacteria, employs a rotary mechanism to consume the energy stored in the electrochemical proton motive force (∆p) and drive the synthesis of ATP from ADP and inorganic phosphate [1,2]. The simplest bacterial ATP synthases consist of eight different subunits arranged in two domains: the hydrophilic F_1_ domain (α_3_β_3_γδε) and the membrane-intrinsic F_O_ domain (*ab*_*2*_*c*_*9–15*_) [2]. Protons enter and exit the membrane domain through subunit *a*, but are also exchanged with the *c-*ring, causing the *c*-ring to rotate. The *c*-ring is connected to the central stalk (γε). As the central stalk rotates it induces conformational changes in the α and β subunits in F_1_, converting ADP to ATP. The peripheral stalk (*b*_*2*_δ) acts as a stator, preventing F_1_ rotating with the central stalk, and thus it maintains a strict coupling between proton translocation and ATP synthesis.

**Fig. 1:**
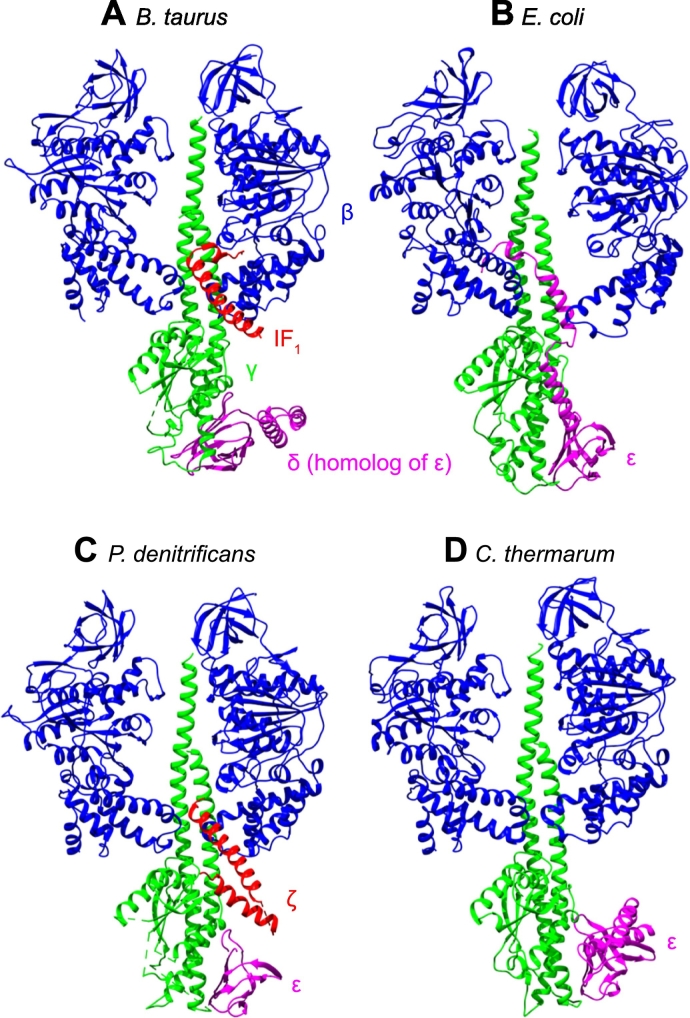
Structures of the F_1_ domains of four ATP synthase enzymes in states with ATP hydrolysis inhibited. For clarity, only two β subunits (blue) are shown with the γ subunit (green), bacterial ε subunit/mitochondrial δ subunit (magenta) and the unique *P. denitrificans* ζ subunit and mitochondrial inhibitor protein IF_1_ (red). (A) The F_1_ domain from *Bos taurus* with a monomeric form (residues 1–60) of the inhibitor protein IF_1_ bound (PDB 2V7Q) [6]. Residues 8–50 of IF_1_ are resolved. (B) The F_1_ domain from *Escherichia coli* with the ε subunit in the inhibitory ‘up’ state (PDB 3OAA) [11]. (C) The F_1_ domain from *P. denitrificans* with the partially resolved ζ subunit and the resolved region of the ε subunit shown; the two α-helices of the ε-CTD are not resolved (PDB 5DN6) [7]. (D) The F_1_ domain from *Caldalkalibacillus thermarum*, which is unable to hydrolyze ATP even with the ε subunit in the ‘down’ state (PDB 5HKK) [17].

ATP synthases are, in principal, reversible enzymes: when the ∆p is low and [ATP]/[ADP] is high, the thermodynamics of the system favor ATP hydrolysis over ATP synthesis, leading to generation of ∆p [3]. Many microorganisms use fermentation as their primary ATP-generating process under anaerobic growth conditions, and use ATP synthase to hydrolyze ATP and produce the ∆p to maintain essential cellular functions [4,5]. However, uncontrolled ATP hydrolysis can rapidly deplete the cellular ATP pool and therefore many organisms have evolved distinct mechanisms to limit the ATP hydrolysis reaction. In mitochondrial ATP synthases the inhibitory factor 1 (IF_1_) inserts into the rotary machinery of F_1_ blocking rotation in the hydrolysis direction [6] (Fig. 1). A similar strategy is thought to be employed by the ζ subunit of the α-proteobacterium *Paracoccus denitrificans* [7,8], and by the C-terminal domain of the ε subunit in some bacteria [[9], [10], [11]] (Fig. 1). A different mechanism has been adopted in chloroplasts where, in the dark, when ∆p is no longer maintained by photosynthesis and conditions favor ATP hydrolysis, a reversible disulfide bond is formed within the γ subunit, fixing it in position and preventing rotation in either direction [12,13]. Finally, in all organisms ATP hydrolysis is inhibited by its products, ADP and/or phosphate, but to varying degrees [[14], [15], [16]].

The species-specific roles of the bacterial ε subunit, and how it regulates ATP hydrolysis are widely debated but poorly understood [18,19]. The ε subunit consists of two domains, an N-terminal flattened β-sandwich and a C-terminal domain (ε-CTD), which consists of two antiparallel α-helices [11,17]. The N-terminal domain is important for connecting the F_1_ and F_O_ domains [20,21] and is required for the assembly and function of the enzyme, whereas, in some organisms at least, the ε-CTD is considered to regulate ATP hydrolysis by interconverting between two different conformations [22,23]. The ‘down’ or hairpin conformation is non-inhibitory and, in organisms such as *Bacillus* PS3, has been proposed to be stabilized by ATP binding [23]. The ‘up’ or extended conformation inhibits hydrolysis by inserting into an αβ interface in the F_1_ domain, blocking rotation (Fig. 1) [11,22,24]. It has been proposed that removal of the ε-CTD from *E. coli* ATP synthase increases ATP hydrolysis rates (from 0.80 to 1.20 μmol min^−1^ mg^−1^) but decreases proton pumping, suggesting that the ε-CTD is further required to maintain coupling between ATP hydrolysis and proton translocation [25]. Conversely, a separate study suggested that proton pumping in *E. coli* ATP synthase is not significantly affected by ε-CTD removal, despite a > 2-fold increase in ATP hydrolysis activity, making it unclear whether the ε-CTD plays a role in coupling [26]. Hydrolysis rates in the isolated F_1_ domain from *Caldalkalibacillus thermarum* (TA2.A1) increased more markedly upon removal of the ε-CTD, from 0.12 to 0.85 μmol min^−1^ mg^−1^ [9]. The crystal structure of the *C. thermarum* F_1_ domain [17] revealed the ε-CTD in an ATP-bound down conformation (Fig. 1). However, mutations to ablate the ATP binding site did not activate ATP hydrolysis and the ε-CTD remained in a down conformation [17], suggesting that ATP binding to the ε-CTD is not involved in regulation. Interestingly, the structure also revealed a magnesium-free ADP molecule bound with a phosphate ion in the β_E_ site in F_1_, which was interpreted as being important for the inhibition of ATP hydrolysis.

Inhibition by Mg-ADP is ubiquitous in all ATP synthases [14,15,[27], [28], [29]]. The mechanism is poorly understood, but it appears that binding of Mg-ADP without phosphate induces a conformational change in the catalytic portion of F_1_, locking in the Mg-ADP and preventing further catalysis [15,27]. Mutational studies of F_1_ from the thermophilic *Bacillus* PS3 further showed that binding of nucleotides to noncatalytic sites in the α subunits affects the entrapment of the Mg-ADP, suggesting a complex cross-talk between catalytic and non-catalytic sites [28,29]. Release of the inhibitory Mg-ADP to activate hydrolysis can be achieved in vitro by three methods: generation of a ∆p; addition of the zwitterionic detergent lauryldimethylamine oxide (LDAO); and addition of oxyanions. Activation by ∆p has been demonstrated in ATP synthases from bovine mitochondria [30], chloroplasts [31] and bacteria such as *Bacillus* PS3 [32], *E. coli* [32,33] and *P. denitrificans* [34,35]. However, the conformation of the ε-CTD also seems to depend on ∆p [36] and it is unclear how these two putative regulatory mechanisms interact. Early studies suggested that the ε-CTD acts against the ∆p-induced release of Mg-ADP [32]. However, there is growing evidence to indicate an opposing role, where inhibition by Mg-ADP and ε-CTD are distinct from each other and compete [[37], [38], [39], [40], [41]]. This is supported by observations that addition of the inhibitor azide, which stabilizes the Mg-ADP inhibited state, favors the non-inhibitory hairpin ('down') conformation of the ε-CTD [38,42] and by the strong inhibition of ATP hydrolysis by Mg-ADP in F_1_ of the *B. subtilis* ATP synthase lacking the ε subunit [40]. Activation by LDAO facilitates the release of entrapped ADP by an unknown mechanism [16,28,43], whereas oxyanions such as sulfite or selenite are proposed to occupy phosphate binding sites in F_1_ and to induce conformational changes to release the Mg-ADP [44,45]. These latter two activation methods are also influenced by the ε-CTD, which again makes it difficult to define the mechanism of activation [41,46,47].

Here, we describe how ATP hydrolysis is regulated in the ATP synthase from *P. denitrificans*. Although it has been reported that it can be activated for ATP hydrolysis by formation of ∆p [34,48] and by addition of oxyanions or LDAO [35,45,49,50], the *P. denitrificans* ATP synthase is commonly considered as fundamentally unidirectional, effectively unable to catalyze in the hydrolysis direction [51]. Biochemical and structural analyses found that it contains the unique subunit ζ, which bears similarity to the mammalian IF_1_ inhibitor protein and was thus assumed to regulate ATP hydrolysis [7,8,52,53]. However, we recently demonstrated that an unmarked genetic deletion of the ζ subunit increased hydrolysis rates only two-fold to 0.026 μmol min^−1^ mg^−1^, well below the expected level of a freely hydrolyzing enzyme [49]. Our results suggested that inhibition by the ζ subunit is not the primary mechanism by which ATP hydrolysis is prevented. Here, we describe two unmarked genetic truncations of the C-terminus of the ε subunit that remove the ε-CTD of the *P. denitrificans* enzyme in both the wild-type strain and the ζ subunit knockout strain. We investigate the rates of ATP hydrolysis in each of the resulting six strains, and how they are all activated by mechanisms releasing Mg-ADP inhibition such as ∆p, LDAO and oxyanions. We thereby elucidate the role of the ε-CTD in *P. denitrificans* ATP synthase and reveal the major role that Mg-ADP plays in governing the ability of the *P. denitrificans* enzyme to hydrolyze ATP.

## 2 Results

### 2.1 Design and creation of ε subunit truncations

**Fig. 2:**
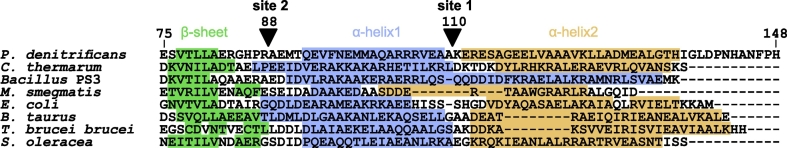
Sequence alignment and secondary structures of the ε-CTD from structurally characterized ATP synthases. The sequences were aligned using Clustal Omega [55] and ordered by their similarity to the ε-CTD from *P. denitrificans*, as calculated by EMBOSS Needle [55]. The α-helices of the ε-CTD of *P. denitrificans* were predicted using JPred4 [56]. All other secondary structures were resolved in structural analyses: *P. denitrificans* 5DN6 [7], *C. thermarum* 5HKK [17], *Bacillus* PS3 6N2Y [10], *Mycobacterium smegmatis* 6FOC [57], *E. coli* 3OAA [11], *B. taurus* 5ARA [58], *Trypanosoma brucei brucei* 6F5D [59] and *Spinacia oleracea* 6FKF [13]. Sites of truncation for the *P. denitrificans* ε-CTD are shown by black triangles.

In order to study the role of the ε-CTD in inhibiting ATP hydrolysis by *P. denitrificans* ATP synthase, the subunit was truncated from the C-terminus, retaining the N-terminal domain that is important for assembly of the intact enzyme [20,21]. The ε-CTD is not resolved in the only available structure [7] (Fig. 1) so secondary structure predictions were performed to select the sites of truncation. The predictions suggest that the *P. denitrificans* ε-CTD consists of two α-helices connected by a short linker, similar to the known secondary structures present in *E. coli* and other homologs (Fig. 2) [11]. Based on the data in Fig. 2, two truncations were designed, one after residue 88 removing both α-helices (ε^Δ88^) and the other after residue 110 removing only the C-terminal α-helix (ε^Δ110^). The truncations were created (see Materials and Methods) in a strain of *P. denitrificans* devoid of a hydrogenase operon [54], unrelated to the ATP synthase, which is referred to here as ‘wild-type’. The same truncations were also created in our ζ-knockout strain (∆ζ, a derivative of the same hydrogenase knockout strain) [49] leading to the variants ∆ζε^Δ88^ and ∆ζε^Δ110^.

**Fig. 3:**
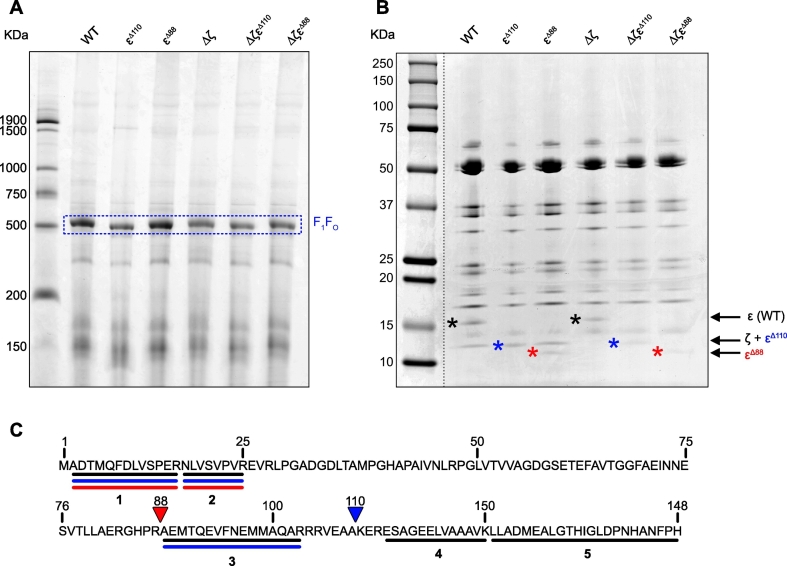
Confirmation of enzyme assembly and ε subunit truncations. (A) BN-PAGE analyses of SBPs from each strain. Proteins were solubilized using *n*-dodecyl β-D-maltoside and the bands corresponding to intact ATP synthase (F_1_F_O_) are indicated. (B) SDS-PAGE of ATP synthase bands excised from the BN-PAGE gel. Wild-type and ε truncations (ε^∆110^ and ε^∆88^) are highlighted by black, blue and red asterisks, respectively. The ζ subunit comigrates with the ε^∆110^ protein. (C) Key tryptic peptides identified by mass spectrometry in bands containing the ε subunits excised from the SDS-PAGE gel. The amino acid sequence for the ε subunit is shown with the key peptides identified (1–5) in the wild-type (black), ε^∆110^ (blue) and ε^∆88^ (red) ε subunits, and in both the ζ knockout and ζ-containing strains. For ions scores see SI Table S1.

All six strains grew similarly under aerobic conditions in both LB medium and a defined succinate medium (SI Fig. S1), showing that they all contain a functional ATP synthase. Blue Native PAGE (BN-PAGE) of detergent-solubilized sub-bacterial particles (SBPs, inverted cytoplasmic membrane vesicles) confirmed that truncating the ε-CTD (as observed previously for the ζ subunit deletion [49]) does not affect assembly of the intact enzyme (Fig. 3A). A clear band representing the fully assembled enzyme was visible in all constructs, consistent with the expected molecular mass (558 kDa in wild-type). The corresponding bands were excised and analyzed by a second dimension of SDS-PAGE to visualize the individual subunits, revealing a similar subunit composition in each case. The only differences were a shift of the ε band in the strains with a truncated ε-CTD and the absence of a band in the ζ region for the ζ knockout strains (Fig. 3B). Finally, the bands belonging to the ε subunits were excised, digested with trypsin and analyzed by mass spectrometry. Two N-terminal peptides (labelled 1 and 2 in Fig. 3C) were detected in all strains. Peptide 3 (residues 88–103), characteristic of the first of the two C-terminal helices, was only detected in the ε^Δ110^ variants, while peptides 4 and 5, characteristic of the second C-terminal helix, were absent in both the ε^Δ88^ and ε^Δ110^ variants (Fig. 3C, SI Table S1). Overall, these data confirm successful truncations of the ε-CTD without disruption of the ATP synthase structure.

### 2.2 Removing the ε-CTD is not sufficient to activate ATP hydrolysis

**Fig. 4:**
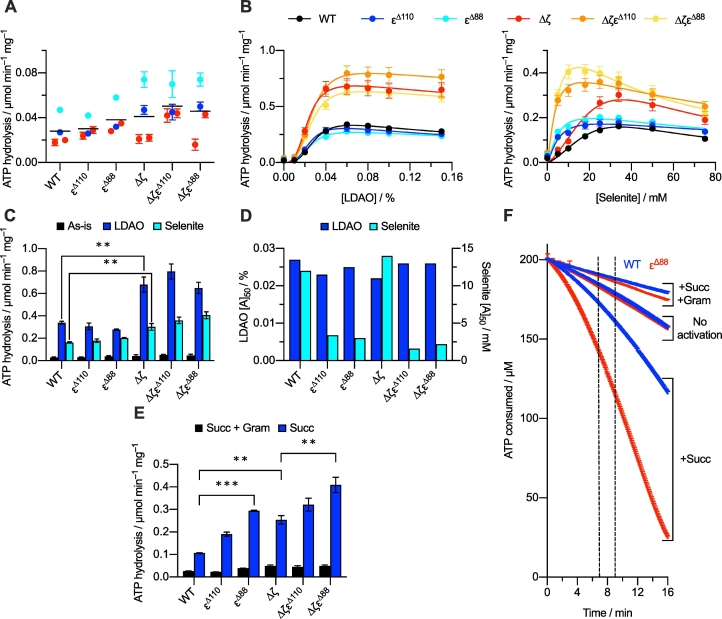
Activation of ATP hydrolysis by *P. denitrificans* ATP synthase in wild-type and variant SBPs. For all panels reporting specific activity, ATP hydrolysis have been normalized to relative ATP synthase content determined by BN-PAGE analyses, and rates were measured using the NADH-coupled assay, in which ATP is regenerated and maintained at near constant concentration (see Materials and Methods). (A) ATP hydrolysis in all six strains. Each point represents the average from three technical replicates with error bars showing the S.E.M. Two separate biological preparations of SBPs are shown (red and blue/cyan). Red points were measured on two separate days and blue/cyan points measured in the presence (blue) or absence (cyan) of 8 μg mL^−1^ of gramicidin. The mean for all data points for each strain is shown by black horizontal lines. None of the differences between strains are statistically significant. (B) ATP hydrolysis in all six strains activated by increasing concentrations of LDAO and selenite. Rates were calculated in the linear region between 100 and 300 s. Curves were fit to the data to guide the eye and determine [A]_50_ values (see Materials and Methods). (C) The maximal rates of ATP hydrolysis for each strain before (black) and after activation by LDAO (blue) or selenite (cyan) taken from panel B. Only the effect of deleting the ζ subunit is statistically significant (two examples of the significance are marked). (D) The [A]_50_ values of activation by LDAO (blue) or selenite (cyan) for each strain. Values were read directly off fitted titration curves in panel B. Only the effects of truncating the ε-CTD on selenite activation are substantial. (E) ATP hydrolysis by wild-type and variants of *P. denitrificans* ATP synthase upon membrane energization by addition of 2.5 mM succinate. Rates were calculated in the linear region between 7 and 9 min, as indicated on panel F. The effects of deleting the ζ subunit and truncating the whole ε-CTD (ε^Δ88^) are both statistically significant (three examples are shown). (F) Examples of kinetic traces monitoring ATP hydrolysis in the wild-type (blue) and ε^Δ88^ (red) strains in the presence/absence of succinate-induced membrane energization (to generate ∆p), and the presence/absence of 8 μg mL^−1^ gramicidin A (to dissipate ∆p). The data for panels B, C and E are mean average data from three technical replicates with error bars showing the S.E.M. Statistical significance was calculated by one-way ANOVA using Tukey's test, **p < 0.01, ***p < 0.001.

The effect of truncating the ε subunit on the rate of ATP hydrolysis was determined in *P. denitrificans* SBPs by using a standard NADH-coupled ATP regenerating assay (see Materials and Methods). All the rates remained low (≤0.074 μmol min^−1^ mg^−1^), even in the absence of both the ζ subunit and the ε-CTD, and none of the differences observed were statistically significant (Fig. 4A). For comparison, ATP hydrolysis in membrane vesicles from *E. coli* and *B. taurus* occurs at 0.38 and 1.24 μmol min^−1^ mg^−1^, respectively, under similar conditions [49]. Thus, like removing the ζ subunit [49], removing the ε-CTD (either independently, or in combination with the ζ subunit) is not sufficient to activate ATP hydrolysis substantially in the *P. denitrificans* ATP synthase. The low rates measured for all strains were close to background levels and appeared to be influenced by the quality of membrane coupling (note the apparent changes upon addition of the uncoupler gramicidin, Fig. 4A). Although both these effects contribute to variability in the data and prevent robust comparisons being made, it is clear that inhibition from the ε-CTD is either very weak or non-existent in *P. denitrificans*. We note that removing the ε-CTD in *Bacillus* PS3 [60] resulted in a clear 5-fold increase in activity, suggesting either that the ε-CTD behaves differently in the two enzymes, or that additional regulatory mechanisms mask the effect of removing it in the *P. denitrificans* enzyme.

### 2.3 Activating ATP hydrolysis with LDAO and oxyanions

Clearly, neither the ζ subunit nor the ε-CTD provide the primary mechanism by which ATP hydrolysis in *P. denitrificans* is prevented. A possible alternative mechanism is inhibition by Mg-ADP, which can be relieved by addition of the detergent LDAO or oxyanions such as phosphate, sulfite or selenite [16,28,43,44]. Thus, LDAO and selenite were titrated against SBPs from all six strains and the corresponding ATP hydrolysis rates were measured (Fig. 4B). For LDAO, maximal rates of ATP hydrolysis were similar for the wild-type, ε^Δ88^ and ε^Δ110^ strains (0.28 to 0.34 μmol min^−1^ mg^−1^, Fig. 4C), nearing the rates observed previously for *E. coli* membrane vesicles [49] — but in the absence of LDAO. All the ∆ζ strains (∆ζ, ∆ζε^Δ88^ and ∆ζε^Δ110^) displayed around two-fold higher rates (0.65 to 0.80 μmol min^−1^ mg^−1^, Fig. 4C) than their ζ-containing counterparts, consistent with previous reports on LDAO-activation of the Δζ strain [49,50]. Only the increases observed upon deleting the ζ subunit were statistically significant so we conclude that truncating the ε-CTD does not affect the extent of activation by LDAO. The substantial rates observed with LDAO suggest it relieves the primary mechanism of inhibition of ATP hydrolysis in *P. denitrificans* ATP synthase. Furthermore, all six strains exhibit similar [A]_50_ values (the concentrations at which the rates are half maximal) for LDAO activation (Fig. 4D), suggesting that LDAO is unlikely to act at a point where the ε-CTD and/or ζ subunit interact with the αβγ interface, as has been suggested previously [47,50].

For the oxyanion selenite (SeO_3_^2−^), the maximal rates obtained are lower than for LDAO, but the activation shows a similar pattern: statistically significant increases in rate are observed for the Δζ strains relative to the ζ-containing strains (Fig. 4B, C) but the small increases observed for the ε-CTD-lacking strains are not statistically significant. Notably, however, markedly lower concentrations of selenite are required to achieve maximal activation in the four strains with truncated ε-CTDs (Fig. 4B, D). The [A]_50_ values for selenite activation decreased from 12.0 mM in the wild-type to 3.4 and 3.0 mM in the ε^Δ110^ and ε^Δ88^ strains, respectively, and the same pattern is replicated in the three Δζ strains (Fig. 4D). Thus, truncating the ε-CTD facilitates activation by selenite, and the different patterns of behavior of LDAO and selenite suggests that they occupy distinct binding sites and hence relieve Mg-ADP inhibition via different routes. Finally, stimulation of ATP hydrolysis with selenite also stimulates proton pumping in all six strains (SI Fig. S2), showing that the activated enzymes remain coupled; the same experiment is not possible with LDAO as, being a detergent, it compromises the integrity of the membrane. These results agree with the most recent findings on the *E. coli* enzyme, where proton pumping is also not significantly affected on removal of the ε-CTD [26].

Our results show that LDAO and oxyanions act predominantly by relieving Mg-ADP inhibition and not by releasing inhibition by the ζ subunit or the ε-CTD: their effects are conserved or enhanced in the absence of ζ and/or the ε-CTD. LDAO is a zwitterionic detergent with no resemblance to any of the substrates. Therefore, it may disrupt one or more subunit interfaces, ‘loosening’ the enzyme structure and assisting in release of inhibitory Mg-ADP, leading to increased hydrolytic activity. Conversely, oxyanions such as sulfite and selenite resemble phosphate, so their mode of activation likely involves them binding to the (nucleotide) phosphate binding sites, either the catalytic sites in the β subunits or the non-catalytic sites in the α subunits, helping to promote ADP release [35,44]. Our results suggest that the ε-CTD may either stabilize an ADP-bound state or destabilize a selenite-bound state, because less selenite is required to attenuate inhibition in the ε^Δ110^ and ε^Δ88^ variants than in the wild-type variant. This is irrespective of the presence of the ζ subunit, the deletion of which exerts a consistent activating effect independent of the LDAO or selenite concentrations. We note that, for selenite, our results differ from results reported previously for the *E. coli* and *Bacillus* PS3 enzymes [[37], [38], [39], [40]]; recently, Milgrom and Duncan proposed distinct pathways for Mg-ADP and ε-CTD inhibition [41] in *E. coli*, consistent with the [A]_50_ value for selenite activation remaining the same in both their wild-type and ε-CTD-lacking *E. coli* membranes (4.7 and 4.4 mM, respectively). It is possible that the roles of the ε-CTD are different in *P. denitrificans* and *E. coli*.

### 2.4 Activating ATP hydrolysis with ∆p

Activation of ATP hydrolysis in SBPs from *P. denitrificans* by formation of a ∆p has been described previously [34,35,48]. Here, we established a ∆p by succinate oxidation (coupled to proton pumping via complexes III and IV) and investigated the effect on ATP hydrolysis in all six variants. Although the observed activation levels are subject to variations in succinate oxidation rates and membrane coupling, membrane energization clearly increased ATP hydrolysis substantially in all strains (Fig. 4E), although activation was slow, taking minutes to reach maximal rates (Fig. 4F). Rates of ATP hydrolysis were also found to increase in the absence of succinate, though much more slowly. However, no activation was observed in the presence of the uncoupler gramicidin, even with succinate present, suggesting that (in the absence of succinate) slow ATP hydrolysis gradually builds a low ∆p, which initiates a positive feedback loop by activating hydrolysis to increase ∆p further, and so on. The activities reported in Fig. 4E were measured between 7 and 9 min, before the NADH required for the coupled assay is depleted in any of the experiments. Importantly, ATP hydrolysis rates in the presence of succinate were increased significantly by both deletion of the ζ subunit and truncation of the ε subunit. In this respect, activation by ∆p is clearly distinct from activation by LDAO and selenite: only for succinate is the extent (rather than susceptibility) of activation increased by truncation of the ε subunit. Thus, although all three modes of activation are considered to act by relieving inhibition by Mg-ADP, they exhibit different characteristics that likely reflect their different activation mechanisms.

### 2.5 ATP synthesis is not compromised by removal of the ε-CTD

**Fig. 5:**
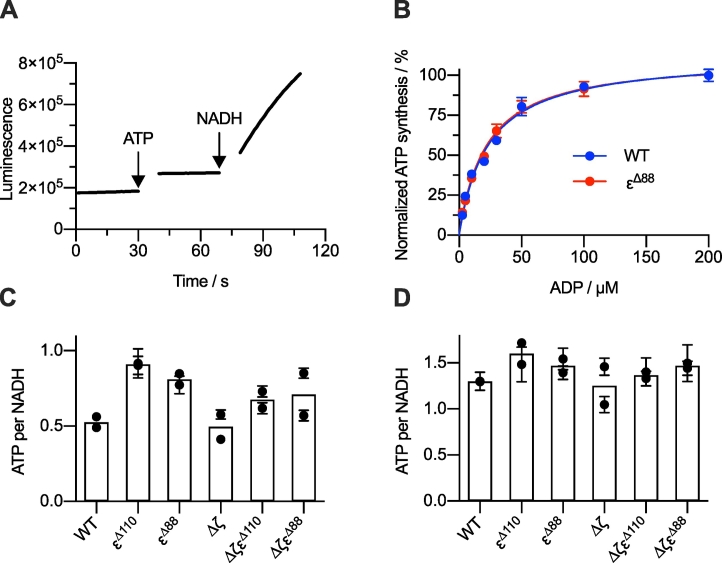
ATP synthesis by SBPs from wild-type and variant *P. denitrificans* strains. (A) Kinetic trace monitoring ATP synthesis by SBPs in real-time from the ε^Δ88^ strain in the presence of 50 μM ADP. The luminescence was calibrated by addition of 1 μM ATP. See Materials and Methods for details. (B) Dependence of the rate of ATP synthesis on ADP concentration for SBPs from the wild-type and ε^Δ88^ strains. The curves are normalized to maximum ATP synthesis rates with *V*_max_ values of 0.775 ± 0.025 (wild-type) and 1.293 ± 0.034 (ε^Δ88^) μmol min^−1^ mg^−1^ and *K*_M_ values of 23.0 ± 2.3 (wild-type) and 21.7 ± 1.8 (ε^Δ88^) μM. The rates are mean averages of three technical replicates ± S.E.M. (C) The number of ATP molecules generated per NADH molecule oxidized for all six strains, measured by the real-time monitoring of ATP synthesis. Initial rates were measured at room temperature in the presence of 50 μM ADP. (D) The number of ATP molecules generated per NADH molecule oxidized for all six strains, measured by a quenched-based ATP quantification assay [54]. Rates were measured at 32 °C in the presence of 1 mM ADP. Data points in panels C and D represent two biological replicates with rates reported as the average of three technical replicates ± S.E.M. All rate comparison between strains are non-significant according to one-way ANOVA using Tukey's test.

Following our conclusion that removing the ε-CTD in *P. denitrificans* ATP synthase promotes the release of ADP inhibition, we asked whether deleting the ε-CTD affects ATP synthesis. To measure ATP synthesis, NADH was added to SBPs to create a ∆p by respiratory chain catalysis, and ATP production was monitored continuously using a luciferase-based assay system (Fig. 5A). Initial synthesis rates were measured in the wild-type and ε^Δ88^ strains at different ADP concentrations. The *K*_M_ values for ADP remained similar at 23.0 ± 2.3 and 21.7 ± 1.8 μM, respectively, suggestive of a similar affinity for ADP (Fig. 5B). ATP synthesis was measured for each strain, under two different conditions, and the number of ATP molecules generated per NADH oxidized was calculated (Fig. 5C+5D). This normalization accounts for variations in the purities of the SBP preparations, and is based on the fact that (within individual preparations) the rate of ATP synthesis depends linearly on the rate of NADH oxidation [61]. In neither condition was the rate of ATP synthesis affected by truncation of the ε-CTD. We note that an increase in ATP synthesis on removal of the ε-CTD has previously been described for both the *E. coli* and *Bacillus* PS3 enzymes [62,63], but here, the differences observed were not statistically significant. We conclude that removal of the ε-CTD does not compromise the ATP synthesis capability of the enzyme.

## 3 Discussion

How ATP hydrolysis is regulated by different mechanisms in the ATP synthases from different species is a key unanswered question in ATP synthase research. The crudest way to stop hydrolysis is simply to prevent rotation mechanically, as implemented in chloroplasts by disulfide bond formation [12,13]. In mitochondria, the inhibitor protein IF_1_ provides a more sophisticated approach by binding like a stick through the spokes of a wheel when the enzyme is rotating in the hydrolysis direction [64]. One current hypothesis is that when the enzyme switches to synthesis and rotation begins in the opposite direction, the inhibitor is ejected. These external mechanisms of regulation are necessary to prevent hydrolysis because the mitochondrial and chloroplast ATP synthases are otherwise excellent catalysts in both directions, catalyzing ATP hydrolysis to drive proton translocation with rates of 400–520 s^−1^ [65,66]. Although similar rates of ATP hydrolysis have been reported for the *E. coli* enzyme [67], many bacterial ATP synthases catalyze hydrolysis with much slower rates, and some of them with essentially no rate at all. The ATP synthase of *P. denitrificans* is one such example, and it was proposed that this is due to its ζ subunit, which resembles IF_1_ [7,8,52,53]. However, it is now clear that the ζ subunit exerts only a moderate effect on the rate of hydrolysis, with its genomic deletion activating low-level catalysis only around two-fold, such that hydrolysis remains very slow [49]. Alternative explanations are thus now being sought, and, based on the role of the ε-CTD in regulating hydrolysis by *E. coli* and *Bacillus* PS3 ATP synthase [10,22,60], we first explored that option here. We found that removing the ε-CTD is not sufficient to activate hydrolysis in *P. denitrificans* (the rate remains very slow with both the ζ subunit and ε-CTD removed), and this argues against direct inhibition by the ε-CTD binding to a catalytic interface in F_1_ during hydrolysis to hinder rotation, as observed in *Bacillus* PS3. Additionally, while the ε-CTD in *Bacillus* PS3 possesses an ATP binding domain that controls its conformation in response to the ATP concentration [23], the *P. denitrificans* ε-CTD contains no such binding site. If there is such a conformational transition in *P. denitrificans* then it may be in response to a change in ∆p, which has also been described for *Bacillus* PS3 [68]. Here, we were unable to identify any direct inhibition from the ε-CTD. However, removal of the ε-CTD made the enzyme more sensitive to activation of ATP hydrolysis by the addition of oxyanions such as selenite and increased the extent of activation upon energization of the membrane. LDAO, oxyanions and ∆p have all been proposed to act by alleviating inhibition by Mg-ADP [16,28,43,44], a universal feature of all ATP synthases on some level, and here we suggest that all our results can be rationalized if they represent different mechanisms for destabilizing or precluding the Mg-ADP-bound inhibitory state. Therefore, we contend that inhibition by Mg-ADP is the primary mechanism by which ATP hydrolysis is prevented in *P. denitrificans*, while the ε-CTD and unique ζ subunit provide only secondary and independent effects, which may only become important under activating conditions where ADP-inhibition is not dominant.

Two models can be envisaged for inhibition of ATP hydrolysis by Mg-ADP. One is that the rate of dissociation for the product ADP is very low (a dominant rate determining step). The other is that the affinity of the enzyme for an inhibitory Mg-ADP, locking it in an inactive state, is very high. However, the first case is less consistent with ADP as the substrate for ATP synthesis (at least in the simplest model where ATP hydrolysis is the exact reverse of ATP synthesis); during ATP synthesis ADP is required to bind ‘back’ into the catalytic site, arguing against slow binding/dissociation kinetics. A high affinity Mg-ADP could be present either in a catalytic site or a non-catalytic, allosteric site. LDAO, oxyanions and ∆p would act by decreasing its affinity — although the molecular mechanisms by which any of these effects occur are currently unclear. We have suggested that LDAO binds at subunit interfaces to loosen the structure and weaken nucleotide binding; it is difficult to probe the effects of LDAO further because it interferes with membrane integrity and therefore compromises studies of proton translocation and ATP synthesis. Oxyanions resemble phosphate, and are likely to compete with the bound ADP. The fact that ∆p is effective in speeding up ATP hydrolysis suggests an important role of ∆p in regulation of ADP-inhibition. However, it is striking that, thermodynamically, ∆p opposes the hydrolysis, proton-pumping reaction, and so ∆p appears to kinetically assist but thermodynamically oppose the same reaction. It is possible that in the cell, where the ∆p varies, the ζ subunit and the ε-CTD may take on greater importance in preventing ATP hydrolysis.

Prior work by Zharova and Vinogradov has previously suggested that the *P. denitrificans* enzyme is inhibited by Mg-ADP, and that ∆p prevents the formation of the Mg-ADP inhibited state [34,48]. It was suggested that, on the collapse of ∆p, phosphate dissociates first from the β subunit, leaving the enzyme trapped in an inactive Mg-ADP inhibited state. Here, we show that removing the ζ subunit and ε-CTD increase ATP hydrolysis significantly in the presence of ∆p (Fig. 4F). While removing the ζ subunit appears to stimulate hydrolysis independently of the mode of activation, the patterns of activation observed upon removal of the ε-CTD are more varied, suggesting a more complex interplay with Mg-ADP inhibition, which is not yet fully understood. Although recent evidence points to a destabilizing/competing role of the ε-CTD on Mg-ADP, at least for the *E. coli* and *Bacillus* PS3 enzyme [[37], [38], [39], [40], [41]], our data suggest that the opposite could be true in *P. denitrificans*. In *P. denitrificans*, the ε-CTD may stabilize Mg-ADP inhibition, as removing the ε-CTD increases the enzyme's sensitivity to oxyanion activation (Fig. 4D) and its activity in the presence of ∆p. It is also possible that the increased sensitivity to oxyanions results from the ε-CTD destabilizing the oxyanion-bound state rather stabilizing Mg-ADP inhibition. Alternatively, both ∆p and oxyanions have been suggested to activate different inhibited states present in the ATP synthase population [35]. It is thus possible that removing the ε-CTD instead results in a change in the distribution of these states, altering the fraction of the enzyme population that is susceptible to activation by ∆p. However, this would imply that a substantial portion of the enzyme population remains inactive even in the presence of a ∆p, the most physiologically relevant activating condition. Further evidence is required to distinguish these possible models and disentangle the different observations (and possible different roles of the ε-CTD) in different organisms.

In the absence of structural data on the *P. denitrificans* ε-CTD, it is not known if its conformation resembles either of the ‘up’ or ‘down’ conformations observed for other ATP synthases such as the *E. coli* ATP synthase [11,69], although an up-like conformation provides a more obvious way for it to influence ADP or oxyanion binding in the F_1_ domain. However, if the ε-CTD is in a defined conformation then it is unclear why it is unresolved in the crystal structure of the *P. denitrificans* ATP synthase. One reason may simply be that the detergent used for solubilization disrupts the ε-CTD or makes it more flexible. Alternatively, the ε-CTD may be mobile and insert itself only when F_1_ begins to rotate during ATP hydrolysis. It would be interesting to solve the structure of the *P. denitrificans* ATP synthase activated for ATP hydrolysis by cryo-EM, as has been done with the ATP synthase from *E. coli*, to define the different catalytic (sub-)states of the rotor and the conformations of the ε-CTD [69].

In summary, inhibition by Mg-ADP is the dominant mechanism by which *P. denitrificans* ATP synthase is prevented from catalyzing ATP hydrolysis. The inhibition is particularly relevant and effective in the absence of ∆p when the driving force for ATP hydrolysis is high. It is possible that in order to support the development of greater biological complexity, ATP synthases have evolved to minimize the strength of ADP-inhibition, to become more reversible and thus more effective at energy conversion. As a result, organisms in which ATP synthase is able to catalyze ATP hydrolysis efficiently have evolved additional external mechanisms to prevent the wasteful hydrolysis reaction, as observed in mitochondria and chloroplasts.

## 4 Materials and methods

### 4.1 Generation of ε subunit truncations

Truncations of the ε subunit were created in the ∆hydrogenase (∆Hy) and ∆hydrogenase∆ζ (∆Hy∆ζ) strains of *P. denitrificans Pd*1222 described by Jones et al., and Varghese et al., respectively [49,54]. Here, we designate the hydrogenase knockout strain as our ‘wild-type’ strain. Homologous recombination was used to create unmarked truncations of the ε subunit by following a similar strategy as described previously for deleting the hydrogenase and ζ subunits. The ε subunit (Pden_3819) is found at nucleotide position 959291–959737 on chromosome 2. Truncation cassettes were designed containing two sequences homologous to regions on either side of the ε-CTD. The first homologous flanking region began at nucleotide position 957857 and extended to positions 959617 or 959551 giving a flanking region of length 1761 and 1695 bp for respective ε^Δ110^ and ε^Δ88^ constructs. Directly following this, the next flanking region began at position 959735, which misses out the ε-CTD but includes the STOP codon (959735–959737) at the end of the ε subunit. The flanking region extended to position 960958 for a total length of 1224 bp. This second flanking region is followed by a kanamycin (*kan*^*R*^) selection marker of 815 bp length. For completeness, regions 959618–959734 and 959552–959734 were deleted in ε^Δ110^ and ε^Δ88^ constructs, respectively. *Eco*RI restriction sites were added to the end of the construct and any *Eco*RI sites within the construct itself were removed by silent mutagenesis. The construct was assembled by GENEWIZ® and then inserted into the *lacZ*-containing pRVS1 suicide plasmid via an *Eco*RI restriction site. The plasmid was transformed into the MFD*pir E. coli* donor strain and conjugated into both the ∆Hy and ∆Hy∆ζ strains. Successful conjugation and first recombination events were selected by using LB-agar plates containing 50 μg mL^−1^ kanamycin. Successful colonies were then plated on X-gal (200 μg mL^−1^), and white colonies were selected as positive for the second recombination event, and screened by PCR amplification of the DNA sequence across the ε subunit to confirm the expected decrease in length of the ε encoding gene. The integrity of the truncated strains was then confirmed by full sequencing of the ATPase operons.

### 4.2 Preparation of sub-bacterial particles (SBPs) from *P. denitrificans*

*P. denitrificans* SBPs were prepared as described by Jones et al.^54^ except that the composition of the cell lysis buffer was adjusted. Briefly, cells were grown aerobically at 30 °C and 225 rpm and harvested by centrifugation at mid-exponential phase (OD_600_ = 2.5–3.0). All subsequent steps were performed at 4 °C and all centrifuge steps at 14000 ×*g*. The cell pellets were resuspended in 10 mM Tris-SO_4_ (pH 7.5 at 4 °C) and 150 mM NaCl, recentrifuged and resuspended in buffer containing 10 mM Tris-SO_4_ (pH 7.5 at 4 °C) and 500 mM sucrose to an OD_600_ of approximately 7.5. Hen egg-white lysozyme was added to the suspension to a final concentration of 250 μg mL^−1^, and the suspension was incubated for 60 min before the digested cells were collected by centrifugation. The pellet was then resuspended in buffer containing 10 mM Tris-SO_4_ (pH 7.5 at 4 °C) and 500 μM MgSO_4_ to lyse the cells. After cell lysis, MgSO_4_ was added to a final concentration of 5 mM, along with a few flakes of bovine pancreatic DNase, and the supernatant was centrifuged twice to remove the cell debris. The SBPs were collected by centrifuging the supernatant for 1 h, the pellet was resuspended in buffer containing 10 mM Tris-SO_4_ (pH 7.5 at 4 °C) and 250 mM sucrose to a concentration of 5–10 mg mL^−1^ and frozen in liquid N_2_ for storage at −80 °C.

### 4.3 Electrophoresis and mass spectrometry

Blue native polyacrylamide gel electrophoresis (BN-PAGE) was performed using NativePAGE Novex 3–12% Bis-Tris gels (Invitrogen) [70]. The protein concentration of SBPs was determined using a standard Pierce™ BCA protein assay (Thermo Fisher Scientific) and SBPs were solubilized at a ratio of 2:1 *n*-dodecyl β-D-maltoside:protein in buffer containing 10 mM Tris-SO_4_ (pH 7.5 at 4 °C) and 150 mM NaCl. Solubilization was performed for 30 min in a Thermomixer comfort at 4 °C with continuous mixing at 700 rpm. Insoluble material was then removed by centrifugation at 30,000 ×*g* for 30 min at 4 °C. Solubilized proteins (10 μg) were loaded into each well, run alongside solubilized bovine mitochondrial membranes for reference, and bands were visualized using Coomassie R250. Gels were imaged in an Epson Perfection V850 Pro scanner and band intensities were compared using the Fiji software [71]. For SDS-PAGE analyses, bands of interest were excised and incubated in loading buffer (0.125 M Tris-HCl (pH 6.8), 20% (w/v) glycerol, 4% (w/v) SDS, 0.005% (w/v) bromophenol blue and 0.1 M DTT) for 30 min at 30 °C, with mixing at 700 rpm in a Thermomixer comfort. The combined proteins from four gel slices were then loaded into each well of a Novex WedgeWell 10–20% tris-glycine gel, run as described previously and visualized using Coomassie R250 [72]. Bands excised from SDS-PAGE gels were digested with trypsin and analyzed by mass spectrometry using an Q-Exactive Plus Orbitrap mass spectrometer as described previously [49,72]. Spectra were assigned to peptide sequences and their originating proteins using the Mascot 2.4 application (Matrix Science Ltd.) with a peptide precursor mass tolerance of 5 ppm and fragment mass tolerance of 0.01 Da and allowing for up to two missed cleavages and variable modifications (methionine oxidation and formation of acrylamide adducts).

### 4.4 Determination of the rate of ATP hydrolysis

ATP hydrolysis was measured at 32 °C in a Molecular Devices SpectraMax Plus 96-well microplate reader using a coupled assay system that oxidizes NADH in response to the production of ADP [73,74]. Assay buffer contained 10 mM Tris-SO_4_ (pH 7.5 at 32 °C), 250 mM sucrose, 2 mM MgSO_4_, 2 mM K_2_SO_4_, 200 μM phosphoenolpyruvate, 200 μM NADH, 50 μg mL^−1^ lactate dehydrogenase from bovine heart, 40 μg mL^−1^ pyruvate kinase from rabbit muscle and 200 μM ATP. Complex I was inhibited with 2 μM piericidin A. For measurements in the presence of a ∆p, SBPs were preincubated with 2.5 mM succinate for 3 min at 32 °C before initiating the hydrolysis reaction. The ∆p was dissipated by addition of 8 μg mL^−1^ gramicidin A [75]. Curve fitting was carried out in GraphPad Prism 8. LDAO and selenite activation curves were fit to an allosteric, sigmoidal model that corrects for substrate/activator inhibition at high activator concentrations and fit the data well. The model used was: Y=V_max_*x^h^/(K_m_^h^ + x^h^(1 + x/K_i_)) with the slope h constrained to a shared value between all measurements and K_m_ and K_i_ to values greater than zero. The fitted curve was used only to guide the eye for determining [A]_50_ values.

For all NADH-coupled assays, the ATP hydrolysis rates have been normalized to the relative amount of ATP synthase content in the batch of SBPs assayed. Three independent solubilizations of each SBP preparation were analyzed and compared on the same gel or across multiple gels. Individual bands from BN-PAGE analyses for each strain were compared to the average band intensity for all strains run on the same gel to determine relative ATP synthase content.

### 4.5 Determination of the rate of ATP synthesis

#### 4.5.1 Real-time ATP monitoring assay

ATP synthesis was monitored continuously using a Promega GloMax 20/20 luminometer with 50 μL reaction volumes in 1.5 mL tubes at room temperature. The assay buffer contained 20 mM Tris-PO_4_ (pH 7.5 at RT), 5 mM MgCl_2_, 100 μM diadenosine pentaphosphate (Ap5A) [76] and a 1:50 dilution of luciferase reagent (ATP Bioluminescence Assay Kit CLS-II, Roche) [77]. Typically, 12 μg mL^−1^ SBPs were assayed at room temperature in the presence of 50 μM ADP [78]. Baseline luminescence and the background rate were recorded for 30 s before 1 μM ATP was added to calibrate the luminescence (see Fig. 5A). Proton pumping by complexes I, III and IV was started by addition of NADH (200 μM) and ATP synthesis was measured continuously. Initial (linear) rates (up to 10–30 s) of ATP synthesis were used. For determination of the number of ATP molecules generated per NADH oxidized, NADH oxidation rates of samples in ATP synthesizing conditions were measured directly at 340–380 nm (ε = 4.81 mM^−1^ cm^−1^) in a Molecular Devices SpectraMax Plus 96-well microplate reader.

#### 4.5.2 Quenched ATP monitoring assay

ATP synthesis was monitored in an assay based of that from Jones et al. [54]. ATP synthesis was measured at 32 °C in a cuvette using a Molecular Devices SpectraMax Plus 96-well microplate reader. Assay buffer contained 10 mM Tris-SO_4_ (pH 7.5 at 32 °C), 250 mM sucrose, 2 mM MgSO_4_, 10 mM KPO_4_, 1 mM ADP, 250 μM Ap5A, 20 units mL^−1^ superoxide dismutase (bovine erythrocytes), 5000 unit mL^−1^ catalase (Corynebacterium glutamicum) and 12 μg mL^−1^ SBPs. The reaction was initiated with 200 μM NADH and the NADH oxidation was monitored spectroscopically. After 1 min, ATP production was monitored by withdrawing and quenching 10 μL aliquots of the reaction mixture into 40 μL of 4% trifluoroacetic acid, followed 20 s later by addition of 450 μL neutralizing buffer (1 M Tris-SO_4_ pH 8.0). Aliquots were withdrawn and quenched at 30 s intervals over a total assay length of 3 min. ATP concentrations in the quenched aliquots were determined using the Roche ATP Bioluminescence Assay Kit CLS II and measured in a Molecular Devices SpectraMax Gemini XPS 96-well microplate.

## Funding

This work was supported by the 10.13039/501100000265Medical Research Council (MC_UU_00015/2 to J.H.) and by the 10.13039/501100001711Swiss National Science Foundation (P2BEP3_181897 to O.B.).

## CRediT authorship contribution statement

O.D.J. created and characterized the mutant ε strains. O.D.J. and O.B. carried out catalytic activity assays. J.H., O.B. and O.D.J. designed and J.H. coordinated the study. O.D.J. and J.H. wrote the manuscript with help from O.B.

## Declaration of competing interest

The authors declare that they have no known competing financial interests or personal relationships that could have appeared to influence the work reported in this paper.

## Appendix A Supplementary data

Supplementary material Image 1
