# Supplementary material for: Regulation of ATP hydrolysis by the ε subunit, ζ subunit and Mg-ADP in the ATP synthase of Paracoccus denitrificans
Source: Biochim Biophys Acta Bioenerg. 2021 Mar 1;1862(3):148355. doi: 10.1016/j.bbabio.2020.148355 (PMC8039183; doi:10.1016/j.bbabio.2020.148355)
Supplement: Supplementary file 1 — Supplementary material [file mmc1.docx]

**Supplementary Information**

**
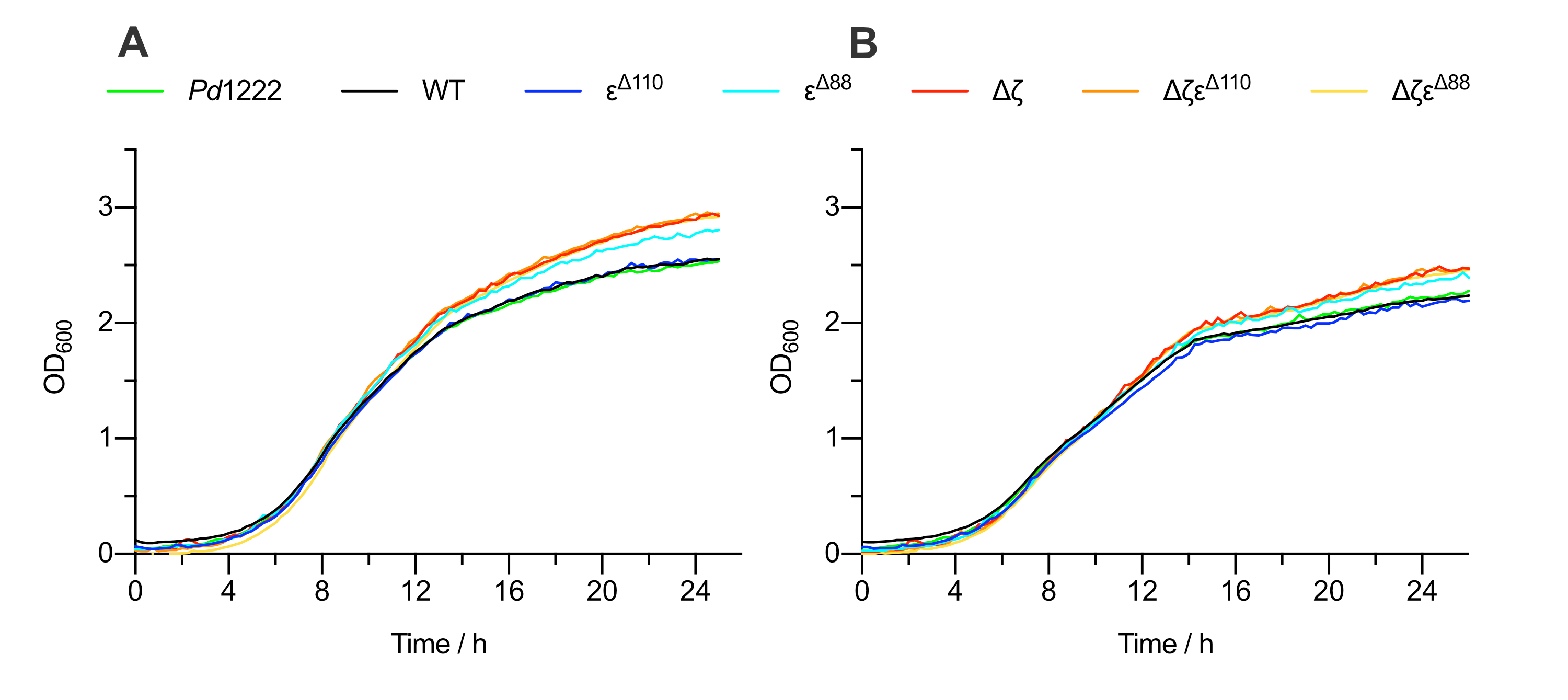
**

**Figure S1.** Comparison of growth curves for *P. denitrificans* cells from the parental *Pd*1222 strain, the ∆hydrogenase strain referred to here as the wild-type, the ∆ζ strain and the ε subunit truncations in both the wild-type and Δζ strains. Cells were grown in 1 mL of either (A) LB or (B) succinate minimal medium at 30 ˚C with 200 rpm orbital shaking in a CLARIOstar *Plus* microplate reader. Optical density was recorded every 15 mins. Experiments were initiated using a freshly grown stationary-phase starter culture added to reach a starting optical density of 0.02 (at 600 nm) and carried out in triplicate. The minimal medium was adjusted to pH 7.2 and contained 50 mM succinate, 9.35 mM NH_4_Cl, 2 mM MgSO_4_, 0.07 mM CaCl_2_, 0.29 mM KH_2_PO_4_, 0.69 mM K_2_HPO_4_, 25.2 mM Na-Hepes, 19.6 μM Na_2_-EDTA, 9 μM FeSO_4_, 0.1 μM MnCl_2_, 0.8 μM CuCl_2_, 1 μM Na_2_MoO_4_ and 2.5 μM ZnCl_2_.

**Figure S2.** Comparison of membrane energization in SBPs from each strain during NADH oxidation or ATP hydrolysis, monitored by the rate of quenching of the fluorescence of 9-amino-6-chloro-2-methoxyacridine (ACMA) at 32 ˚C [75]. Reactions were initiated by addition of either NADH (500 μM) or ATP (1 mM) to 1 mL of 50 μg mL^–1^ SBPs stirred in buffer containing 10 mM MOPS (pH 7.5), 50 mM KCl, 1 mM MgCl_2_, 0.5 μM ACMA and 100 nM valinomycin, with or without 10 mM selenite. Membranes were de-energized by addition of 40 mM NH_4_Cl after 4 min.

| **Peptide Label** | **Peptide** | **Ion Score / 45** | | | | | |
| --- | --- | --- | --- | --- | --- | --- | --- |
|  |  | **WT** | **ε^∆110^** | **ε^∆88^** | **∆ζ** | **∆ζε^∆110^** | **∆ζε^∆88^** |
| **1** | ADTMQFDLVSPER | 73 | 92 | 106 | 106 | 86 | 92 |
| **2** | NLVSVPVR | 37 | 39 | 37 | 37 | 39 | 39 |
| **3** | AEMTQEVFNEMMAQAR | 57 | 80 | – | 66 | 78 | – |
| **4** | ESAGEELVAAAVK | 53 | – | – | 78 | – | – |
| **5** | LLADMEALGTHIGLDPNHANFPH | 57 | – | – | 50 | – | – |

**Table S1.** Summary of Orbitrap analyses (Figure 3C) on ε subunit bands excised from the SDS-PAGE gel shown in Figure 3B. The Mascot ion score for each reported peptide is shown in comparison to the p < 0.05 ions score cut-off (45 for all peptides).

| **Strain** | **ATP synthase content relative to average band intensity on BN-PAGE analyses** | | |
| --- | --- | --- | --- |
|  | **Preparation 1 (Fig. 4A+B+C)** | **Preparation 2 (Fig. 4A+E)** |  |
| WT | 1.295 ± 0.043 | 1.074 ± 0.023 |  |
| ε^∆110^ | 0.946 ± 0.084 | 1.251 ± 0.058 |  |
| ε^∆88^ | 1.368 ± 0.023 | 0.895 ± 0.009 |  |
| ∆ζ | 0.667 ± 0.066 | 0.970 ± 0.065 |  |
| ∆ζε^∆110^ | 0.744 ± 0.062 | 0.974 ± 0.087 |  |
| ∆ζε^∆88^ | 0.882 ± 0.066 | 0.836 ± 0.070 |  |

**Table S2.** Relative ATP synthase content in SBPs prepared for all strains from two separate batches. Three independent solubilizations of each SBP preparation were analyzed and compared on the same gel or across multiple gels. Individual bands from BN-PAGE analyses for each strain were compared to the average band intensity for all strains run on the same gel. Values shown are mean averages of three independent solubilizations of each SBP preparation ± S.E.M.
